# PEEK in Fixed Dental Prostheses: Application and Adhesion Improvement

**DOI:** 10.3390/polym14122323

**Published:** 2022-06-08

**Authors:** Biyao Wang, Minghao Huang, Pengrui Dang, Jiahui Xie, Xinwen Zhang, Xu Yan

**Affiliations:** 1 Liaoning Provincial Key Laboratory of Oral Diseases, The VIP Department, School and Hospital of Stomatology, China Medical University, No. 117 North Street Nanjing Road, Shenyang 110002, China; cmu2015353201@163.com (B.W.); dangpengruitg@163.com (P.D.); jiahuixie777@163.com (J.X.); 2 Liaoning Provincial Key Laboratory of Oral Diseases, Center of Implant Dentistry, School and Hospital of Stomatology, China Medical University, No. 117 North Street Nanjing Road, Shenyang 110002, China; 2020121580@stu.cmu.edu.cn

**Keywords:** polyetheretherketone, properties, adhesion, bonding, fixed dental prosthesis

## Abstract

Polyetheretherketone (PEEK) has been widely applied in fixed dental prostheses, comprising crowns, fixed partial dentures, and post-and-core. PEEK’s excellent mechanical properties facilitate better stress distribution than conventional materials, protecting the abutment teeth. However, the stiffness of PEEK is not sufficient, which can be improved via fiber reinforcement. PEEK is biocompatible. It is nonmutagenic, noncytotoxic, and nonallergenic. However, the chemical stability of PEEK is a double-edged sword. On the one hand, PEEK is nondegradable and intraoral corrosion is minimized. On the other hand, the inert surface makes adhesive bonding difficult. Numerous strategies for improving the adhesive properties of PEEK have been explored, including acid etching, plasma treatment, airborne particle abrasion, laser treatment, and adhesive systems.

## 1. Introduction

Polyetheretherketone (PEEK), a member of the polyaryletherketone (PAEK) family, has recently emerged as a promising alternative material for fixed dental prostheses. PEEK is a high impact polymer material that is made from fluorine benzene ketone, hydroquinone, and sodium carbonate or potassium carbonate, dissolved in diphenyl sulfone. PEEK consists of an aromatic nucleus linked by ketone and ether groups, providing it with a superior chemical stability that seems to be a double-edged sword [1,2]. Regarding its advantages, PEEK resists high-temperature thermal stress (melting point, 343 °C) without significant degradation, shows low water solubility (0.5%), and is able to minimize biocorrosion within body fluid, thus avoiding the release of metal ions that can trigger cytotoxicity, allergy, and inflammation [3]. Because of this, PEEK not only prolongs the lifespan of a prosthesis, but also protects the abutment teeth and other adjacent tissues [4]. Furthermore, because of its aromatic chemical structure, PEEK is resistant to the gamma and electron beams that are commonly used for sterilization [5]. In addition, PEEK is radiolucent and generates few imaging artifacts, providing significantly better performance than zirconia and metal alloys [6]. In terms of its disadvantages, PEEK has an inert and poorly adhesive hydrophobic surface (surface contact angle, θ at 65°) that is an important obstacle to its wider application in fixed prosthodontics [7]. Improvement strategies are summarized later in this article.

PEEK exhibits excellent mechanical properties, including a low Young’s modulus (3 to 4 GPa), which, compared to metal alloys (110 to 130 GPa), is closer to that of human cortical bone (14 GPa) [8]. Given its deformability, PEEK can provide more balanced stress distribution, limiting stress-shielding when serving as an implant [9] and lowering the risk of root fracture when used for post-and-core restoration [10]. However, when compared with conventional materials, the stiffness of PEEK (tensile strength 110 MPa) is not sufficient to sustain the load-bearing stress [11]. To address this, as shown in Table 1, PEEK can be blended with glass fibers or carbon fibers in order to enhance its mechanical strength [12,13]. Notably, glass fiber reinforced- and carbon fiber reinforced-PEEK (GFR-PEEK, CFR-PEEK) exhibit an even more similar Young’ s modulus (12 GPa and 18 GPa) to human bone and dentin, along with greater flexural strength (170 MPa) and better color stability, providing more favorable outcomes [12,13,14]. The thermal conductivity of PEEK is 0.29 W/mK, which is lower than that of zirconia and protects the abutment teeth from temperature fluctuations in the mouth [14]. PEEK has a lower wear rate (0.9 ± 1.1 mm^3^/MC) than metal and its alloys (1.6 ± 2.0 mm^3^/MC), which should lead to significant improvement for crown restorations [15]. Additionally, PEEK has a tensile property comparable to that of the tooth and considerably low density (1.31 g/cm^3^), which can result in favorable stress distribution for lightweight framework restorations [15].

PEEK exhibits superior biocompatibility [16]. It is nonmutagenic and nontoxic to human gingival fibroblasts and osteoblasts both in vitro [17,18] and in vivo [3]. There is currently no evidence to suggest that PEEK induces allergic immune responses in humans, and PEEK can be considered as an alternative restoration material for people allergic to metal [15]. An assay comparing PEEK with titanium alloy (Ti6Al4V) and zirconia showed no significant difference in pro-inflammatory cytokine gene expression [19]. Moreover, PEEK exhibited significantly lower susceptibility to biofilm formation than Ti6Al4V [19]. Similar biocompatibility results have also been reported for GFR- and CFR-PEEK [20,21,22]. PEEK has shown favorable osseointegration and antibacterial properties, which should further promote its desirability as a promising alternative material for oral implantology [23,24,25].

PEEK is a metal-free material with a gray color. It provides aesthetic improvement for implantology when compared with metal and its alloys [26], whereas in comparison to zirconia it fails to achieve a satisfactory aesthetic outcome when serving as a fixed dental prosthesis [27,28,29]. PEEK requires veneering with composite resin to improve this aesthetic effect [29], but the inert surface of PEEK makes bonding between PEEK and composite veneers difficult [7]. Bonding between PEEK and resin-based luting materials, including Panavia V5 (0.8 ± 0.4 MPa), RelyX Ultimate Resin Cement (5.2 ± 1.3 MPa), G-CEM Link Force (3.7 ± 1.4 MPa), and Super-Bond C&B (8.2 ± 1.3 MPa), fails to reach the accepted shear bond strength (SBS; ≥ 10 MPa) [30], and PEEK requires various modification methods for improved bonding, as summarized in the later part of this article. This article reviews the application of PEEK in fixed dental prostheses and discusses adhesion improvement strategies. CFR-PEEK and GFR-PEEK referenced in this article are 30% fiber-reinforced unless otherwise specified.

**Table 1:** The mechanical and physical properties.

Mechanical and Physical Properties	PEEK	GFR-PEEK	CFR-PEEK	Ti6Al4V	Cortical Bone	Dentin
Specific gravity (g/cm^3^)	1.31	1.51	1.41	4.34	1.92	
Young’s modulus (GPa)	3–4	12	18	110–130	14	18.6
Tensile strength (MPa)	110	97	131	976	104–121	104
Tensile modulus of elasticity (GPa)	4.3	6.9	7.6	113	13.6–28.3	
Tensile elongation (at break) (%)	40	2	5	6–10	1–3	
References	[7,21]			[21,22]	[31]	[7]

## 2. Fixed Dental Prostheses

**Figure 1:**
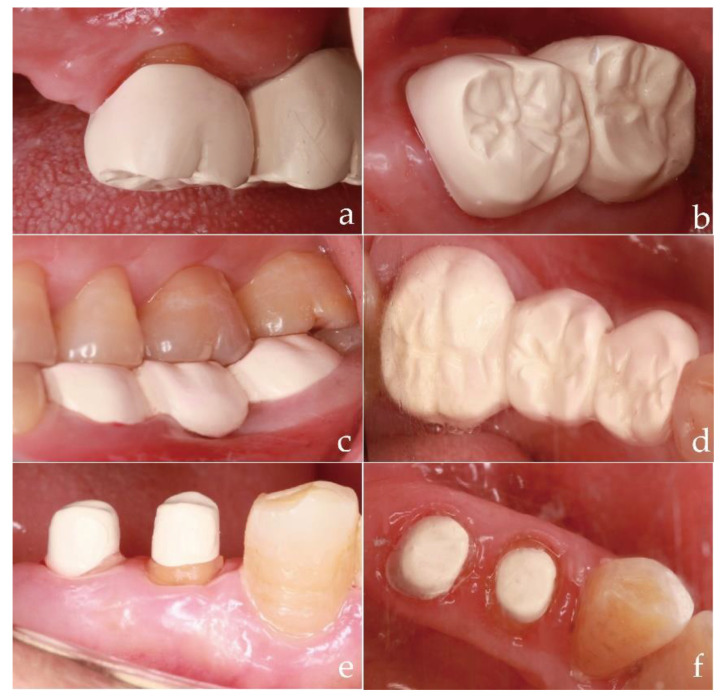
Clinical photographs of PEEK prostheses: (**a**) Frontal view of PEEK crowns; (**b**) Occlusal view of PEEK crowns; (**c**) Frontal view of PEEK fixed partial dentures; (**d**) Occlusal view of PEEK fixed partial dentures; (**e**) Frontal view of PEEK post-and-core; (**f**) Occlusal view of PEEK post-and-core.

The success of a fixed dental restoration depends on three key factors: biomechanical behavior (wear resistance and fracture resistance), marginal fit, and aesthetics, generating extremely strict demands for the restoration material. Zirconia has become a popular alternative to metal in fixed dental prostheses, known for its excellent aesthetics [32,33,34]. More importantly, zirconia exhibits better wear resistance than metal and alloys [35]. PEEK is proposed as a promising alternative material to zirconia because of its superior mechanical properties. PEEK is also significantly less abrasive than zirconia [36]. Figure 1 shows clinical photographs of fixed dental prostheses made of PEEK.

This study was conducted in accordance with the Declaration of Helsinki, and the protocol was approved by the Medical Ethics Committee of the Hospital of Stomatology of China Medical University (2022; No. 7). Patients provided written informed consent to publish case details and any images.

### 2.1. Crowns

Regular mastication and progressive erosion result in unavoidable wear of the crown, and the crown material must possess considerable wear resistance [37]. Numerous authors have examined the wear resistance of PEEK crowns. Abhay et al. [38] and others [39,40] have reported that zirconia crowns exhibit greater displacement resistance than PEEK crowns, but are also more abrasive, and although PEEK showed greater susceptibility to displacement compared to zirconia, it also shows a more balanced distribution of stress through deformation because of its much lower elastic modulus (3 to 4 GPa vs. 210 GPa) [38]. Regarding wear, an in vitro study comparing polymethylmethacrylate (PMMA), PEEK, and silicate ceramic (SiO_2_) crowns demonstrated the pivotal role of crown geometry in crown preservation [41]. The PEEK crown exhibited increasing material loss along with the elevation of the cusp inclination, while showing minimum material loss in comparison to PMMA and SiO_2_ after thermal loading [41].

Regarding fracture resistance, PEEK exhibits superior flexural strength (140 to 170 MPa) compared to conventional materials, protecting restorations from bulk fractures [6,42]. Shetty et al. found that crowns with PEEK coping exhibited much greater strength than crowns with zirconia coping [43], and thermocycling had minimal effect on fracture resistance. Finite element analysis (FEA) has indicated that PEEK crowns and porcelain fused to metal crowns have similar stress distribution in dentin [13]. Tekin et al. reached a different conclusion, however, as a veneered PEEK crown reduced the stress concentration in dentin, post, and composite core in comparison to the porcelain fused to metal crown, while increasing the stress concentration in the cement layer of the post and crown [44]. Recent FEA modelling for implants with insufficient alveolar bone support has examined connected crowns, which can alleviate the stress concentrations at the margin of the crown and tooth [45]. Notably, PEEK is recommended as a long-term provisional crown material in cases where other auxiliary treatments are planned. When compared with polylactic acid and PMMA in vitro, PEEK exhibits the lowest marginal and internal gap values and the greatest fracture resistance [46]. Besides, Sulaya et al. conducted a one-year in vivo longitudinal pilot study that assessed the prosthetic performance of PEEK crowns and found that 90% were satisfactory under the modified Ryge Criteria, with a low incidence of fracture [47].

Precise margins are crucial to successful crown restorations, with failure resulting in adhesive dissolution, dentin hypersensitivity, secondary caries, and periodontitis. Crowns with PEEK coping had better margin fit and internal adaptation than crowns with zirconia coping, and both were clinically acceptable [48]. Variations in manufacturing techniques exert a significant effect on margin precision. Pressed PEEK exhibited a larger marginal gap than computer-aided design (CAD)- and computer-aided manufacturing (CAM)-milled PEEK, both of which stayed within the clinical acceptance limit [14]. As yet, only a few studies have been dedicated to examining the differences among various fabrication technologies, and further research is required.

### 2.2. Fixed Partial Dentures

Stress distribution, fracture resistance, and fracture pattern are primary considerations for fixed partial dentures (FPDs) [49]. The Young’s modulus (3 to 4 GPa) of PEEK is lower than that of CoCr alloys (220 GPa) and zirconia (220 GPa) [50,51]. Given this advantage, when the occlusal force load is at the pontic, PEEK provides stress absorption for the abutment teeth, protecting them from fracture [52]. Campaner et al. used FEA to compare the mechanical performance of three-unit FPDs of acrylic resin, resin composite, and PEEK [53], and found that in the PEEK prosthesis, the connectors provided greater stress distribution than the other parts of the prosthesis. As to the cement layers, PEEK displayed the lowest strength of the cervical margin, indicating that PEEK could alleviate the stress concentration in FPDs. However, the highest strengthening of the occlusal region was also observed in PEEK.

Rodríguez et al. examined the potential of PEEK as an alternative FPD material [54] along with various fracture patterns and reported that CoCr registered the highest fracture values after thermocycling, followed by PEEK (3132 N) and zirconia; all were within the clinically acceptable range [55]. Moreover, in another study, Stawarczyck et al. [56] reported a lower fracture value (1383 N) of an uncemented three-unit milled PEEK FPD and noted that deformation appeared to start at 1200 N. Stawarczyck’s group also studied the effect of the fabrication technique on fracture resistance in PEEK FPDs [57] and found that granulate pressed PEEK had a lower fracture value (1738 N) than milled PEEK (2354 N). In terms of the fracture pattern, pressed PEEK pellets and milled PEEK had fracture at the pontic without deformation, while deformation without fracture was observed in pressed PEEK granules [57]. Regarding the underlying mechanism, Niem et al. found that PEEK in a three-unit FPD had a superior capacity to absorb fracture energy via elastic deformation preceding rupture based on its favorable flexural modulus and on respective stress-strain curves marked by increased strain values [52]. The size of the connector is also thought to have a critical role in the fracture resistance of PEEK FPDs. Among the few studies that have concentrated on this, some [54,57] support a connector size of 16 mm^2^ while others [56] have advocated smaller dimensions (7.36 mm^2^, 11.3 mm^2^). Other factors in FPD fracture include the presence or absence of veneer, aging, abutment models, and so on. Even with the varying degrees of difference in the design of the above-mentioned studies, PEEK can still be regarded as a viable alternative material for FPDs.

In terms of its clinical utility for FPDs, Rauch et al. have noted that PEEK requires less fabrication time and is lighter than zirconia, and although zirconia has exhibited a better aesthetic result than veneered PEEK, both are aesthetically acceptable [6]. PEEK FPDs provide satisfactory clinical outcomes when assessed by modified Ryge Criteria and the California dental assessment system [58]. Only 5% of PEEK FPDs failed because of de-bonding, while remaining restorations were maintained without fracture, and 10% showed marginal discoloration, but marginal adaption exhibited no significant change over one year.

Cekic–Nagas et al. compared the load bearing capacity of inlay-retained FPDs fabricated from PEEK vs. other resin-based materials [59] and found that PEEK had the highest load-bearing capacity and could be considered as an alternative to fiber reinforced composite materials. They and others [58] have found that the majority of fractures of inlay-retained PEEK FPDs occur at the connector. Tasopoulos et al. have recently published a case report describing a successful restoration of an inlay-retained PEEK FPDs [60]. Additional clinical trials are necessary to evaluate the long-term restorative quality.

### 2.3. Post-and-Core

Post-and-core material requires high fracture and fatigue resistance, accurate matching with the morphology of the root canal, and more importantly, a Young’s modulus similar to dentin (18.6 GPa) [61]. The elastic modulus of the post material plays a key role in the stress distribution within dentin, subsequently affecting the fracture performance of the restoration and the teeth [13]. Post materials with a Young’ s modulus closer to that of dentin usually generate favorable stress distribution, with high stress at the post and low stress at the weakened root and post–dentin interface [61]. Cast metal alloy posts and zirconia posts—which have much higher elastic moduli than dentin—generate concentrated stress at the root, which may result in the fracture of the root, while the posts remain intact [62,63]. Fiber-reinforced composite (FRC) posts exhibit more balanced stress distribution, and while the risk of root fracture is lower, the posts are more easily fractured [64]. Nevertheless, because of their excellent mechanical behavior, FRC posts have become the most commonly used material for post-and-core restoration, although there are still some disadvantages. The prefabricated FRC post cannot match the morphology of the natural root canal and requires a specially calibrated drill for canal preparation that may increase the depletion of dentin and the thickness of cement, subsequently raising the risk of root fracture and post debonding [65]. Recent results have shown that PEEK shows better aesthetic behavior than metal alloys and is comparable to FRC when used as post-and-core material; its low elastic modulus (3 to 4 GPa) is comparable to that of dentin (18.6 GPa), as are the elastic moduli of GFR-PEEK (12 GPa) and CFR-PEEK (18 GPa) [13].

FEA consistently confirms the potential of PEEK as an alternative material to FRC or glass fiber in post-and-core restoration. In terms of prefabricated posts, PEEK and glass fiber posts show similar intensity and stress distribution when trialed with an occlusal load [61], and PEEK posts display more favorable stress distribution and failure patterns compared to glass fiber and titanium posts in various structures of the restoration and teeth whether under mechanical or thermal stress [66]. Similarly, in comparison to the glass fiber post, the prefabricated PEEK post reduced the stress concentration within the post, post cement, and composite core, while exhibiting no significant effect within dentin [44]. Carbon fibers and glass fibers can be blended with PEEK to not only increase the stiffness of PEEK, but also to provide a more similar elastic modulus to dentin [67], and CFR-PEEK posts showed the lowest von Mises stress in dentin in comparison to FRC, GFR-PEEK, and polyetherketoneketone posts [13]. Moreover, the maximum stress occurred in the CFR-PEEK posts, and the finding that the stress was lower at the dentin–post interface suggests a protective effect conveyed by the similar elastic modulus [13].

Regarding the influence of the PEEK manufacturing technique, FEA is useful for predicting the mechanical behavior of PEEK in post-and-core restorations and for evaluating the accuracy of PEEK fabricated by different methods. The work of Lalama et al. has predicted higher accuracy of heat-pressed PEEK posts in comparison to CAD/CAM PEEK posts [68].

The superior properties of PEEK as post-and-core material are also evidenced in vitro and in vivo. PEEK posts showed the highest fracture resistance in comparison to polymer infiltrated ceramic (PIC) posts and FRC posts, but teeth had a less favorable fracture result with PEEK than with FRC [65]. PEEK posts exhibited a significantly lower fracture load than nickel-chromium (NiCr) alloy posts while presenting similar fracture resistance to nano-ceramic composites posts and fiberglass posts [69]. Özarslan et al. reported maximum fracture resistance in glass fiber posts, followed by zirconia posts and PEEK posts, and the fracture load of PEEK posts displayed no significant difference when restored with different size root canals [10]. Most failures of PEEK posts resulted from the decementation of post and core and were repairable [10,69]. Sugano et al. tested PEEK in flared root canals in a bovine tooth model but found that PEEK posts showed poor mechanical performance in comparison to glass fiber posts in the restoration of flared root canals [70]. PEEK is growing in popularity among clinicians as a post-and-core material because of its superior aesthetic and mechanical properties. Zoidis et al. have reported on a PEEK post-and-core restoration of a maxillary lateral incisor that was performed at a comparatively lower cost and had a satisfactory outcome [71]. Altogether, accumulating evidence has demonstrated the potential of PEEK to serve as a post-and-core material, but whether PEEK can increase the long-term survival of the teeth and restoration requires additional study.

### 2.4. Other Fixed Dental Prostheses

In addition to common fixed restorations, PEEK has also been tested in vitro for possible use in endocrowns and inlays. Because of decementation, PEEK endocrowns had the lowest retention force in comparison to infiltrated ceramic, partially stabilized tetragonal zirconia, and lithium disilicate ceramic endocrowns [72]. Although PEEK failed to provide sufficient retention, it showed a positive failure pattern, in which the tooth was protected from fracture [72]. When tested as inlay material, CAD/CAM and milled PEEK have also exhibited satisfactory fracture resistance in comparison to direct resin filling [73].

In conclusion, because of the deformability related to its lower elastic modulus, PEEK can provide favorable stress absorption for abutment teeth, adjacent tissues, and the cementation layer in fixed dental prostheses when compared with metal alloys and zirconia [74]. PEEK not only protects the abutment teeth and the cortical bone, but it also decreases the incidence of de-bonding, which contributes considerably to its good success [54]. The mechanical strength of PEEK does not match that of the conventional materials, which can lead to fracture of the PEEK itself [75]. To address this, glass fibers, carbon fibers, and other particles can be used to reinforce PEEK and to obtain a more perfect balance between elasticity and strength [76]. Unfortunately, GFR- and CFR-PEEK exhibit a worse aesthetic property for fixed dental prosthesis, and it is difficult to reach a satisfactory aesthetic outcome even with composite resin veneers [76]. Additionally, whether the accumulating deformation would result in a restoration misfit requires further in vitro and in vivo trials.

## 3. Strategies for Improving Adhesion

The superior mechanical properties of PEEK can be offset by its aesthetic limitations. PEEK requires composite veneering to enhance its aesthetic properties. However, PEEK has an inert surface that makes adhesion difficult, and this is an important hindrance to its potential for widespread application in prosthetics. Numerous techniques have been tested for improving the adhesion of PEEK, including acid etching, plasma treatment, airborne particle abrasion, laser treatment, and adhesive systems.

### 3.1. Acid Etching

Accumulating evidence has demonstrated that sulfuric acid etching can significantly improve the SBS of PEEK. Much attention has been devoted to discovering the ideal acid concentration and etching duration (Table 2). In terms of concentration, 98% sulfuric acid has been associated with better surface roughness values (Ra, 0.74 ± 0.25 μm) and SBS (27.36 ± 3.95 MPa) compared to a control group (Ra, 0.04 ± 0.02 μm and SBS, 1.75 ± 0.66 MPa), and was therefore considered an ideal concentration for the surface modification of PEEK [77]. Further study revealed that 98% sulfuric acid etching created porous and permeable surfaces in PEEK, resulting in better adhesion [78,79,80]. The duration of etching with 98% sulfuric acid has a significant effect on the adhesive behavior of PEEK, and the optimal duration varies according to the manufacturing method. Zhang et al. suggest that the appropriate etching duration for printed PEEK is 30 s because the highest SBS (27.90 ± 3.48 MPa) was achieved at 30 s, while for milled PEEK, the ideal duration was <120 s (SBS, over 29 MPa) [78]. On the other hand, Ma et al. have recommended 5 min as an ideal duration for 98% sulfuric acid etching because it results in lower wettability (5 min θ at 115.3 ± 9.9°) vs. the untreated surface (5 min θ at 92.9 ± 3.2°) while maintaining compatibility, and an intact porous structure under scanning electron microscopy (SEM), with less residual acid [79]. Together, these results suggest that further research will be needed to establish an optimal etching duration for PEEK, which may also provide a theoretical guidance for PEEK modification. The adoption of high-concentration sulfuric acid etching for PEEK surface modification may also be limited, in spite of the excellent improvement of bonding performance, because of the risk of corrosive injury to the mucosa [80]. Besides sulfuric acid, piranha solution and hydrofluoric acid etching have also been reported to enhance the bonding performance of PEEK, but both exhibited unsatisfactory outcomes [81,82,83].

Recent studies indicate that combined methods can have a synergistic effect for improving bonding strength of PEEK. While acid etching improves the SBS of PEEK, acidic adhesive conditioning (pH = 2.5) failed to form favorable adhesion regardless of the processing time (Ra, 0.06 μm, *p* < 0.05) [84]. However, 98% sulfuric acid exhibited a synergistic effect with the acidic adhesive, significantly enhancing the surface roughness, (from 1.05 ± 0.59 at 0 min to 1.26 ± 0.51 μm at 5 min) and bond strength of PEEK (from 4.95 ± 2.86 MPa, 0 min to 21.43 ± 5.00 MPa, 5 min), which increased with the adhesive conditioning time [84]. Conversely, Adem et al. concluded that combined treatment offers no benefit [7] based on the finding that a sulfuric acid-treated group exhibited the highest SBS (13.43 ± 1.42 MPa) in comparison to an airborne particle abrasion + sulfuric acid group (11.72 ± 1.69 MPa), an airborne particle abrasion group (6.43 ± 1.05 MPa), and an untreated group (5.39 ± 1.36 MPa); there was no significant difference between the airborne particle abrasion group and the untreated group, which supported the assertion by Adem that surface morphology has a more pivotal role in adhesion improvement than surface roughness [7].

Sulfuric acid etching considerably increases the SBS of PEEK, but the residual sulfuric acid is proven to trigger damage to human cells [84]. A variety of post-treatment strategies for eliminating the residual sulfuric acid are reported. Zhao et al. found a more unfavorable compatibility of a water immersion group resulting from the residual acid in comparison to a water immersion + acetone rinsing group [85], and NaOH was demonstrated to be an effective strategy to neutralize residual sulfuric acid [86]. Recently, Ma et al. reported that there is no significant difference in efficacy of NaOH immersion, acetone immersion, and hydrothermal immersion in elimination of residual acid, compatibility, and maintaining the porous surface; all may be recommended for the post-treatment of sulfuric acid etched PEEK [79].

### 3.2. Plasma Treatment

Plasma treatment—comprised of helium (He), argon (Ar), oxygen (O), hydrogen (H), nitrogen (N), and their mixed plasma—has shown great promise as an excellent surface modification method for PEEK (Table 3). Plasma treatment is a quick and effective method for improving the SBS of PEEK which only modifies the physical and chemical properties of the PEEK surface within a thin layer [87]. Plasma treatment creates patches on the PEEK surface; the quantity, size, and depth of the patches can be increased over time, and, subsequently, improve the bonding performance [87]. The modifying effect also depends on the energy density of the chosen plasma treatment [88]. Plasma treatment is a safer and simpler method when compared with concentrated acid etching, and because there is little chemical byproduct, post-treatment is not required [88].

The effect of He plasma modification of PEEK remains controversial. Okwa et al. treated fiber reinforced PEEK with He plasma, which significantly increased the surface energies and generated OH functional groups [36]. CFR- and GFR-PEEK also showed higher wettability than a control, resulting from the chemical bonding within the interface, after He plasma modification (CFR-PEEK, θ at 37.2 ± 2.6°; GFR-PEEK, θ at 37.3 ± 4.2° vs. CFR-PEEK, θ at 88.6 ± 0.9°; GFR-PEEK, θ at 74.4 ± 3.0°) [36]. However, Schmidlin et al. and others have reported that He plasma treatment fails to enhance the SBS of ceramic-filled PEEK [89].

Zhou et al. have reported satisfactory adhesion with Ar plasma-treated PEEK, noting that surface changes including cracks, grooves, and deposits were associated with significantly higher SBS (4.0 ± 0.2 MPa) compared to an untreated surface (0.0 ± 0.0 MPa) [81]. Lu et al. observed that PEEK exhibited obvious patches under SEM after Ar, air, and Ar-air plasma treatments [88]. Bötel et al. found that both O plasma- and Ar/O plasma-treated PEEK exhibited better bonding behavior (O, 28.69 ± 4.20 MPa; Ar/O, 24.48 ± 3.22 MPa) than untreated PEEK (18.25 ± 5.15 MPa), and suggested that a 35-min O plasma treatment appears to be the optimal method to improve the SBS of PEEK [90], whereas in another study conducted by Fu et al., O, H, and H/O (2:1) mixed plasma-treated PEEK exhibited no significant change in surface roughness and hydrophilicity when compared to untreated PEEK [91].

**Table 3:** The application of plasma treatment for the improvement of PEEK adhesion behavior.

Strategies	Microscopic Analyses (SEM or AFM Images)	Mean Roughness Values (Ra, μm)	Wettability Assays, Surface Contact Angle (θ, ◦) Mean Values	Shear Bond Strength (SBS, MPa)	Reference, Author, Year
H, O, H/O (1:1) plasma treatment		After 30 min, no significant difference in Ra (O, 0.40 ± 0.07 μm; H, 0.42 ± 0.07 μm; H/O, 0.43 ± 0.06 μm; untreated, 0.41 ± 0.07 μm)	After 10 min, θ become stable: H plasma (41.67 ± 1.15°) < O plasma and H/O plasma (almost 0°)		Fu et al., 2021 [91]
He plasma treatment 1 min for CFR-PEEK and GFR-PEEK in distilled water	In treated C-PEEK and G-PEEK, some pieces of broken adhesive resin were visible along the polishing streaks.		CFR-PEEK: a higher wettability (37.2 ± 2.6°) than control (88.6 ± 0.9°) GFR-PEEK: a higher wettability (37.3 ± 4.2°) than control (74.4 ± 3.0°)	Treated CFR- and GFR-PEEK exhibited significantly higher SBS than control	Okwa et al., 2020 [36]
N, Ar, O, Air plasma treatment 10 min	The surface of plasma treated PEEK transformed to a polar surface	An average surface roughness value of 1.01 ± 0.21 μm after polishing		N (10.04 ± 1.84 MPa) > Ar (9.56 ± 1.35 MPa) > Air (9.27 ± 1.33 MPa) > O (8.59 ± 1.64 MPa) > Untreated (5.38 ± 2.90 MPa)	Younis et al., 2019 [92]
O_2_ plasma and Ar/O_2_ (1:1) plasma 35 min treatment for unfilled PEEK veneered with composite (Gradia)		O_2_ plasma treated (0.76 ± 0.21 μm) > untreated (0.75 ± 0.14 μm); Ar/O_2_ plasma treated (0.68 ± 0.21 μm) < untreated (0.79 ± 0.22 μm)	Ar/O_2_ plasma treated (θ at 2.8 ± 1.3°) < O_2_ plasma treated (θ at 0.0 ± 0.0°)	O_2_ plasma treated (28.69 ± 4.20 MPa) > Ar/O_2_ plasma treated (24.48 ± 3.22 MPa) > untreated (18.25 ± 5.15 MPa)	Bötel et al., 2018 [90]
O_2_ plasma and Ar/O_2_ (1:1) plasma 35 min treatment for 20% TiO_2_-filled PEEK veneered with composite (Gradia)		O_2_ plasma treated (2.1 ± 0.78 μm) > untreated (2.08 ± 0.89 μm); Ar/O_2_ plasma treated (2.86 ± 0.21 μm) < untreated (3.13 ± 0.15 μm)	Ar/O_2_ plasma treated (θ at 2.0 ± 1.6°) < O_2_ plasma treated (θ at 0.0 ± 0.0°)	Ar/O_2_ plasma treated (31.54 ± 3.49 MPa) > O_2_ plasma treated (30.38 ± 5.56 MPa) > untreated (17.31 ± 1.93 MPa)	Bötel et al., 2018 [90]

Abbreviation: SEM, scanning electron microscope; FTIR, Fourier transform infrared spectroscopy spectra; AFM, atomic force microscopy; H, hydrogen plasma; O, oxygen plasma; H/O, hydrogen/oxygen; CFR-PEEK, carbon fiber reinforced-PEEK; GFR-PEEK, glass fiber reinforced-PEEK; He, helium plasma; Ar, argon plasma; N, nitrogen plasma; EUV, extreme ultraviolet; Gradia, veneer composite (GC GRADIA^®^, GC Europe, Leuven, Belgium).

Besides O, H, He, and Ar plasma treatments, N plasma treatment can provide markedly enhanced adhesiveness to PEEK. Younis et al. reported that N plasma treatment resulted in higher PEEK SBS (10.04 ± 1.84 MPa) than Ar (9.56 ± 1.35 MPa), air (9.27 ± 1.33 MPa), and O (8.59 ± 1.64 MPa) plasma treatment, all of which were significantly higher than the SBS in the untreated group (5.38 ± 2.90 MPa) [92]. In addition, all of the groups generated favorable fracture patterns resulting from decementation [92]. Similar results are reported for amorphous, semi-crystalline, and mineral-filled semi-crystalline PEEK [93]. In a nutshell, plasma treatment can be considered as an effective surface modification method for enhancing bonding between PEEK and composite resin veneer.

### 3.3. Sandblasting

Abrasion by sandblasting can increase the surface roughness and wettability of PEEK, subsequently improving the bonding between PEEK and veneering composites (Table 4). Gouveia et al. have reported that treatment of PEEK with 110 μm particles for 15 s at 0.2 MPa results in better bonding strength vs. untreated PEEK [27]. Sandblasting, in combination with other surface modification methods, seems to achieve an even better outcome. Taha et al. found that the combination of sandblasting (50 mm for 15 s at 0.25 MPa) with ER:YAG laser treatment or O plasma treatment resulted in much better SBS (22.0 ± 1.3 MPa; 21.2 ± 0.8 MPa) than sandblasting alone (17.4 ± 2.4 MPa) [94].

Although a majority of studies to date have supported the efficacy of sandblasting, there are a few that have disagreed. Adem et al. found no significant difference in SBS between untreated PEEK (5.39 ± 1.36 MPa) and PEEK treated with 50-μm particles for 10 s at 2 MPa (6.43 ± 1.05 MPa) and reported that the bonding performance (SBS) after sandblasting was much worse than the bonding obtained after 1-min 98% sulfuric acid etching (13.43 ± 1.42 MPa) [7]. Parkar et al. have also reported that PEEK exhibited the best SBS following 1-min 98% sulfuric acid treatment (7.52 ± 1.20 MPa) in comparison to alumina particles sandblasting (3.91 ± 0.59 MPa), which showed better performance than synthetic diamond particle sandblasting (SBS, 2.27 ± 0.39 MPa) [95].

The use of silica-modified alumina particles in sandblasting is an innovative surface modification method that promotes both mechanical and chemical connection between PEEK and the veneering composite, thereby more effectively enhancing the bonding strength [96]. Spyropoulos et al. reported that silica-modified sandblasting (30 μm at 0.28 MPa for 15 s plus silane agent) resulted in better bonding strength (24.1 ± 13.0 MPa) for 20% ceramic filled PEEK than non-modified sandblasting (110 μm, 15 s, 2 MPa) (SBS, 15.2 ± 6.8 MPa) [96]. However, in a recent study conducted by Tosun et al., neither silica-coated sandblasting nor simple sandblasting (50 μm, 15 s, 0.28 MPa) resulted in a significant increase of PEEK SBS [97]. Moreover, Çulhaoğlu et al. found that simple sandblasting (50 μm, 15 s, 0.28 MPa) provided sufficient SBS (10.81 ± 3.06 MPa) for PEEK, whereas SBS after silica-modified sandblasting (30 μm, 15 s, 0.3 MPa) is lower (8.07 ± 2.54 MPa) [98], which is in agreement with the results of Caglar’ s group [99]. In Çulhaoğlu’s experiments, 98% sulfuric acid etching for 1 min exhibited the highest SBS (15.82 ± 4.23 MPa), followed by Yb:PL laser irradiation (5 W, 250 ms frequency) (11.46 ± 1.97 MPa) and then silica-modified sandblasting methods [98].

**Table 4:** The application of sandblasting for the improvement of PEEK adhesion behavior.

Strategies	Microscopic Analyses (SEM or AFM Images)	Mean Roughness Values (Ra, μm)	Wettability Assays, Surface Contact Angle (θ, °) Mean Values	Shear Bond Strength (SBS, MPa)	Reference, Author, Year
Sandblasting (S, 50 µm), Er:YAG laser treatment (L), oxygen plasma treatment (P), and their combination (PS, LS)	S: grooved fissured surface structure L and P: shallower irregular surfaces PS and LS: the most irregular surfaces	LS (2.9 ± 0.1 μm) and PS (2.7 ± 0.1 μm) > S > L (1.3 ± 0.1 μm) and P (1.4 ± 0.1 μm) > Untreated	LS (θ at 6.9 ± 0.7°) and PS (θ at 4.9 ± 0.2°) > S (θ at 8.8 ± 0.6°) > L (θ at 19.6 ± 0.8°) and P (θ at 21.5 ± 2.2°) > Untreated (θ at 34.6 ± 2.2°)	LS (22.0 ± 1.3 MPa) and PS (21.2 ± 0.8 MPa) > S (17.4 ± 2.4 MPa) > L (10.1 ± 1.2 MPa) and P (12.4 ± 0.7 MPa) > Untreated (8.3 ± 0.6 MPa)	Taha et al., 2022 [94]
Sandblasting (S, 50 μm, at 0.28 MPa for 15 s); Silica-modified sandblasting (SS, 30 μm, at 0.28 MPa for 15 s)		No significant increase of Ra values after various treatment		No significant increase of SBS values after various treatment	Tosun et al., 2022 [97]
110 µm alumina particles, 98% sulfuric acid etching, 10–20 µm synthetic diamond particles.	Alumina particles: increased roughness; Acid etching: dissolved the surface; Synthetic diamond particles: Failed to penetrate deep into the surface	98% sulfuric acid (2.106 ± 0.186 μm) > alumina particles (1.706 ± 0.160 μm) > synthetic diamond particles (1.101 ± 0.167 μm) > Untreated (0.147 ± 0.024 μm)		98% sulfuric acid (7.52 ± 1.20 MPa) > alumina particles (3.91 ± 0.59 MPa) > synthetic diamond particles (2.27 ± 0.39 MPa) > Untreated (−)	Parkar et al., 2021 [95]
Silica-modified sandblasting (SS, 30 μm, at 0.3 MPa for 15 s); Sandblasting (S, 50 μm, at 0.28 MPa for 15 s); Acetone treatment (99% for 60 s); Sulfuric acid etching (A, 98% for 60 s); Yb:PL laser irradiation (L, at 5 W, 4 Hz for 30 s).		Yb:PL laser (2.85 ± 0.20 μm) > Sandblasting (2.26 ± 0.33 μm) > Acetone (0.54 ± 0.17 μm) or Untreated (0.53 ± 0.15 μm) > Silica-modified sandblasting (0.42 ± 0.03 μm) > Sulfuric acid (0.35 ± 0.14 μm)	Silica-modified sandblasting (θ at 48.4 ± 6.28°) > Acetone (θ at 70.19 ± 4.49°) or Sulfuric acid (θ at 76.07 ± 6.61°) > Untreated (θ at 79.67 ± 4.97°) > Sandblasting (θ at 84.83 ± 4.56°) and Yb:PL laser (θ at 103.6 ± 4.88°)	Sulfuric acid (15.82 ± 4.23 MPa) > Yb:PL laser (11.46 ± 1.97 MPa) > Sandblasting (10.81 ± 3.06 MPa) > Silica-modified sandblasting (8.07 ± 2.54 MPa) > Acetone (5.98 ± 1.54 MPa) or Untreated (5.09 ± 2.14 MPa)	Çulhaoğlu et al., 2020 [98]

Abbreviation: SEM, scanning electron microscope; FTIR, Fourier transform infrared spectroscopy spectra; AFM, atomic force microscopy; Er:YAG, erbium-doped yttrium aluminum garnet laser.

In conclusion, sandblasting shows promise as an easy, safe, and efficient chairside surface modification method for PEEK, although some results differ. The grain size of the aluminum oxide particles and the duration and pressure of abrasion are determinants of the efficiency of sandblasting for improving the bonding performance of PEEK [100]. Because relatively few studies to date have focused on the effect of these factors, PEEK sandblasting techniques require further study.

### 3.4. Laser Treatment

Numerous studies have also been dedicated to laser treatment for improving the adhesion between PEEK and veneering composites, including carbon dioxide (CO_2_) laser, erbium-doped yttrium aluminum garnet (Er:YAG) laser, neodymium-doped yttrium aluminum garnet (Nd:YAG) laser, potassium titanyl phosphate (KTP) laser, and neodymium-doped yttrium orthovanadate (Nd:YVO_4_) laser (Table 5). However, the effect of laser treatment remains controversial.

CO_2_ laser treatment was not successful in creating mechanical interlocking, resulting in little significant improvement of SBS for PEEK, GFR-PEEK, or CFR-PEEK; the adhesive performance of CFR-PEEK was diminished by the CO_2_ laser treatment, whereas GFR-PEEK displayed no significant change [101]. In another study, conducted by Jahandideh et al., CO_2_ laser treatment resulted in a lower SBS (10.6 ± 1.9 MPa) than Er:YAG laser (14.4 ± 1.7 MPa), but both treatments were associated with significantly enhanced bonding strength of PEEK (untreated PEEK, 7.7 ± 1.8 MPa) [102]. Taha et al. have reported that the favorable effect of the Er:YAG laser treatment (SBS, 10.1 ± 1.2 MPa) is improved when combined with sandblasting (SBS, 22.0 ± 1.3 MPa) [94]. Notably, Ates et al. reached the opposite conclusion about PEEK singly treated with Er:YAG laser (SBS, 6.03 ± 1.04 MPa), which failed to have significant effect on SBS (control group, 6.35 ± 1.21 MPa) [103]. This is consistent with another study reported by Caglar’ s group [99]. However, the potential of the combination of Er:YAG laser with sandblasting (SBS, 12.09 ± 2.08 MPa) or silica-modified sandblasting (SBS, 13.14 ± 1.45 MPa) to improve the adhesion performance of PEEK should not be overlooked [103].

**Table 5:** The application of laser treatment for the improvement of PEEK adhesion behavior.

Strategies	Microscopic Analyses (SEM or AFM Images)	Mean Roughness Values (Ra, μm)	Shear Bond Strength (SBS, MPa)	Reference, Author, Year
Er:YAG, Nd:YAG, and KTP lasers (3 W, 20 Hz for 30 s)	Er:YAG: rougher surfaces without any discernable defects; Nd:YAG: regular and deep pores with distinct pore borders and a relatively rough surface; KTP: carbonization on surfaces		Nd:YAG (16.35 ± 0.63 MPa) > Er:YAG (14.29 ± 0.49 MPa) > KTP (11.3 ± 0.41 MPa) > Untreated (8.09 ± 0.55 MPa)	Ulgey et al., 2021 [104]
Er:YAG laser (1.5 W, 20 s) and CO_2_ laser (4 W, 50 s).			Er:YAG (14.4 ± 1.7 MPa) > CO_2_ (10.6 ± 1.9 MPa) > Untreated (7.7 ± 1.8 MPa)	Jahandideh et al., 2020 [102]
100-μm deep, 150-μm deep, and 200-μm deep Nd:YVO_4_ laser groove treatments	Nd:YVO_4_ laser: a surface lattice pattern with regular grooves and undercuts	200-μm (19.9 ± 1.7 MPa) > 150-μm (19.6 ± 1.6 MPa) > 100-μm (15.9 ± 1.8 MPa) > Untreated (0.5 ± 0.1 MPa)	200-μm (15.0 ± 5.3 MPa) > 150-μm (14.4 ± 4.8 MPa) > 100-μm (13.2 ± 5.4 MPa) > Untreated (4.5 ± 2.9 MPa)	Tsuka et al., 2019 [80]
Laser ablation with 200-μm holes spaced 400 μm apart (D2E4); laser ablation with 200-μm holes spaced 600 μm apart (D2E6); sulfuric acid etching; laser ablation (D2E4) followed by acid etching	CO_2_ laser: good quality and reproducible holes on surfaces, but the resin cement did not penetrate the holes; Sulfuric acid etching: increase roughness; Combination: acid etching smoothed the surface of the samples, decreasing the number of pores and irregularities		PEEK: Sulfuric acid > D2E4 or D2E6 > Combination; GFR-PEEK: D2E4 or D2E6 > Sulfuric acid; CFR-PEEK: D2E6 > Sulfuric acid	Henriques et al., 2018 [101]
Untreated group (C); Sandblasting (S); Silica-modified sandblasting(SS); Er:YAG laser (L); LS; LSS	C and SS: relatively smooth surfaces and minimal irregularities; L: irregular surface with deeper and narrow pits; S, LS and LSS: irregularities with larger but not deeper valleys and pits	LSS (θ at 2.31 ± 0.52°) > LS (θ at 2.20 ± 0.23°) > L (θ at 1.79 ± 0.29°) or S (θ at 1.58 ± 0.15°) > SS (θ at 1.31 ± 0.25°) or C (θ at 1.03 ± 0.11°)	LSS (13.14 ± 1.45 MPa) > LS (6.35 ± 1.21 MPa) or SS (12.07 ± 2.82 MPa) > S (10.97 ± 2.88 MPa) > L (6.03 ± 1.04 MPa) or C (6.35 ± 1.21 MPa)	Ates et al., 2018 [103]

Abbreviation: SEM, scanning electron microscope; FTIR, Fourier transform infrared spectroscopy spectra; AFM, atomic force microscopy; Er:YAG, erbium-doped yttrium aluminum garnet laser; Nd:YAG, neodymium-doped yttrium aluminum garnet laser; KTP, potassium titanyl phosphate laser; CO_2_, carbon dioxide; CFR-PEEK, carbon fiber reinforced-PEEK; GFR-PEEK, glass fiber reinforced-PEEK; Nd:YVO_4_, Neodymium-doped yttrium orthovanadate laser.

Elsewhere, Nd:YAG laser resulted in the highest SBS (16.35 ± 0.63 MPa), followed by Er:YAG laser (14.29 ± 0.49 MPa) and then KTP laser (11.3 ± 0.41 MPa); all had significant effect in improving adhesion (Untreated PEEK, 8.09 ± 0.55 MPa), and PEEK exhibited better bonding performance than zirconia after laser treatment [104]. Nd:YVO_4_ laser groove treatment can enhance the SBS not only between PEEK and veneering composite resin, but also between PEEK and various resin-based luting agents, and the improvement of SBS is directly related to the depth of penetration of the Nd:YVO_4_ laser (200-μm, 15.0 ± 5.3 MPa > 150-μm, 14.4 ± 4.8 MPa > 100-μm, 13.2 ± 5.4 MPa > Untreated, 4.5 ± 2.9 MPa) [80].

### 3.5. Adhesive Systems

Various combinations of surface modification methods and adhesive systems have been advanced to improve the bonding strength of PEEK. Resin luting cement, comprising universal resin cements and self-etch resin cements, has provided promising adhesion improvements for PEEK. Sole application of resin based luting cement often fails to provide sufficient SBS for PEEK. It is typically accepted that SBS should not be lower than 10 MPa [97]. Tsuka et al. tested PEEK bonding with resin-based luting materials including Panavia V5 (0.8 ± 0.4 MPa), RelyX Ultimate Resin Cement (5.2 ± 1.3 MPa), G-CEM Link Force (3.7 ± 1.4 MPa), and Super-Bond C&B (8.2 ± 1.3 MPa) and found that SBS failed to reach 10 MPa with any of them and that the majority of the failure modes resulted from decementation [30]. This finding was consistent with those reported by other groups [95,105]. Consequently, Tsuka et al. have combined these resin-based luting agents with a variety of the surface modification methods mentioned above in an attempt to improve the bonding strength of PEEK [80]. Additional studies have shown that combining resin luting cement with sulfuric acid etching is an excellent strategy for enhancing PEEK’s bonding behavior, with universal resin cements (RelyX ARC and Variolink II) showing better bonding behavior than the self-adhesive resin cement (Clearfil SA Cement) regardless of the duration of the sulfuric acid etching. Bunz et al. found that after long-term simulated aging, treatment with Scotchbond universal group resulted in the highest SBS (7.82 MPa) for air-abraded PEEK, followed by Luxatemp Glaze & Bond (4.78 MPa) and SR Nexco Connect (4.55 MPa) or iBond Universal (4.52 MPa), while the SBS for PEEK and the composite resin decreased significantly during the artificial aging period regardless of the adhesive system [105]. Self-etch resin cement (Multilink N) exhibited better bonding strength (7.52 ± 1.20 MPa) in comparison to resin-modified glass ionomer cement (RelyX Luting 2, SBS: 3.85 ± 0.36 MPa) owing to its bonding with the functional groups in PEEK, and among the subgroups treated with self-etch resin cement, combination with 98% sulfuric acid etching provided the most effective outcome [95].

Alongside various luting cements, bonding primer has also provided improvement of PEEK bonding behavior. A satisfactory bonding strength can be achieved by Visio.link because of its specific composition of pentaerythritol triacrylate (PETIA) in solution, MMA monomers, and additional dimethacrylates, which causes micro-interlocking between resin cement and PEEK and increases the SBS of PEEK [99]. Caglar et al. found that PEEK conditioning with Visio.link provided higher SBS in comparison to PEEK with no adhesive treatment (12.54 ± 2.19 MPa vs. 5.58 ± 0.38 MPa); the adhesive performance was further optimized by combination of Visio.link and sandblasting (SBS, 19.86 ± 2.52 MPa) [99]. Evidence confirming the positive effect of Visio.link for PEEK adhesion continues to accumulate. Zhang et al. treated PEEK with 98% sulfuric acid and then coated it with Visio.link; subsequent cross-sectional SEM imaging indicated that the primer had penetrated the etched pores, and the pore depth increased over the etching time, contributing to the enhancement of micromechanical bonding to the resin cement and ultimately improving the SBS [78]. Elsewhere, Kurahashi et al. reported that when combined with Palapress Vario (an autopolymerizing resin adhesive), ceramic primer treatment (Clearfil Ceramic Primer Plus) initially showed no significant effect on the improvement of SBS (3.55 ± 1.14 MPa) vs. control group (3.19 ± 1.06 MPa); however, when combined with silica-modified sandblasting (Rocatec), the ceramic primer treatment resulted in higher SBS (15.32 ± 1.80 MPa) vs. single application of Rocatec alone (12.31 ± 2.10 MPa) [106]. SE Bond is a water-based primer that has also been found suitable for adhesion of hydrophobic and chemically inert surfaces [107], which quite caters to the properties of PEEK. The hydrophilic primer can penetrate into the porous surface of PEEK, thereby contributing to improved SBS. Zhou et al. found that SE Bond combined with Clearfil AP-X self-etching adhesive gave better bonding strength when compared to the RelyX Unicem phosphate monomer luting cement, and they attributed the difference to the hydrophilic primer [81].

In conclusion, a single application of luting cement often fails to provide satisfactory SBS for PEEK, but the performance improves when the luting cements are combined with appropriate bonding primers and surface modification methods. The available body of research is still insufficient, requiring further study to establish an optimal combination for the adhesion of PEEK.

## 4. Conclusions

As described in this article, because of its excellent mechanical, chemical, biocompatibility, and aesthetic properties, PEEK is regarded as a promising alternative to conventional materials for fixed dental prostheses. PEEK prostheses have exhibited comparable or better clinical performance than metal or zirconia. However, PEEK also has some disadvantages when applied in fixed prosthodontics. Because of its grey color, PEEK fails to achieve the aesthetic effect of zirconia unless it is veneered with composite resin; however, the inert and hydrophobic surface of PEEK makes PEEK bonding with composite resin and abutment teeth difficult. This is a known obstacle to wider adoption of PEEK over conventional prosthetic materials. A variety of techniques for surface modification and improvement of the adhesive properties of PEEK, including acid etching, plasma treatment, airborne particle abrasion, laser treatment, and adhesive systems, are described in this report.

## 5. Future Perspective

As noted, neither lone application of luting cement, nor any single surface modification method, has provided satisfactory adhesive behavior for PEEK [30,105]. This indicates that additional effort should be dedicated to determining the best combination of luting cements and pretreatment methods for improved the bonding performance of PEEK. Few of the surface modification methods that are currently available for PEEK exhibit the safety and operability required for regular clinical practice [84,91,97,104], and much of the available research so far has concentrated on fixed dental restoration, with the bonding behavior of PEEK examined only in vitro or via FEA. More effort should be dedicated to clinical trials in order to fully assess the long-term performance.

The restoration and bonding behavior of PEEK is also known to depend on the fabrication technique, whether 3D-printed, milled, or heat-pressed. The fabrication method affects the mechanical properties of PEEK and has also been associated with restoration outcomes [42,68,73,108]. It remains unclear as to which fabrication method is best for PEEK in prosthetic dentistry, and this is another area that requires further development. It is known that the superior performance exhibited by PEEK in fixed restorations can be further improved by adjusting the properties of PEEK via glass- or carbon-fiber reinforcement (GFR-PEEK, CFR-PEEK) [21,109], particle filling (nano-sized silica, titanium dioxide, nano-hydroxyapatite, amorphous magnesium phosphate) [110,111,112,113,114,115], and coating (titanium, methyl methacrylate, polymethyl methacrylate, polydopamine) [67,116,117]. Some of these materials have also been shown to improve the bonding strength of PEEK [67,113,117]. So far, relatively few studies have concentrated on the application of these novel materials in prosthetics and adhesion improvement, which provides a new platform for investigation.

## Figures and Tables

**Table 2:** The application of acid etching for the improvement of PEEK adhesion behavior.

Strategies	Microscopic Analyses (SEM or AFM Images)	Mean Roughness Values (Ra, μm)	Wettability Assays, Surface Contact Angle (θ, °) Mean Values	Shear Bond Strength (SBS, MPa)	Reference, Author, Year
70, 80, 85, 90, 98% sulfuric acid for 60 s	Formation of broader and deepen pores with increasing concentration	Ra elevated with increasing concentration (from 0.04 ± 0.02 to 0.74 ± 0.25 μm)		SBS enhanced with increasing concentration (from 1.75 ± 0.66 to 27.36 ± 3.95 MPa)	Chaijareenont et al., 2018 [77]
98% sulfuric acid etching and acidic adhesive for 0, 1, 3, 5 min	Well-distributed multi-scale pores and pits over the entire surface	Ra elevated over time (from 1.05 ± 0.59 to 1.26 ± 0.51 μm)	Higher wettability (θ at ~55°) vs. the untreated surface (θ at ∼65°)	SBS enhanced over time (from 4.95 ± 2.86 MPa to 21.43 ± 5.00 MPa)	Escobar et al., 2021 [84]
98% sulfuric acid etching (A, for 1 min); sandblasting abrasion (S, 50 μm, at 2 MPa for 10 s).	A: Sponge-like, complex fiber network characterized surface S: Irregular rough surface Combination: agglomeration of alumina particles inside the pores	Sandblasting (1.37 ± 0.28 μm) > combined (0.78 ± 0.26 μm) > sulfuric acid (0.73 ± 0.20 μm) > untreated (0.29 ± 0.10 μm)		Sulfuric acid (13.43 ± 1.42 MPa) > Combination (11.72 ± 1.69 MPa) > sandblasting (6.43 ± 1.05 MPa) or untreated (5.39 ± 1.36 MPa)	Adem et al., 2021 [7]
98% sulfuric acid etching for 0, 5, 30, 60, 90, 120, 300 s	Etched pores were broadened and deepened over time.			Printed PEEK: the highest SBS (27.90 ± 3.48 MPa) was achieved at 30 s. Milled PEEK: SBS showed no significant difference from 5 to 120 s (over 29 MPa) while decreased at 300 s.	Zhang et al., 2021 [78]
98% sulfuric acid etching for 0.5, 1, 3, 5, 7 min	Formation of a 3D porous network that become more complex over time. 5 min: an intact structure with micro- to nano-scale features 7 min: the porous structure tended to be dissolved		Higher wettability (5 min θ at 115.3 ± 9.9°) vs. the untreated surface (θ at 92.9 ± 3.2°)		Ma et al., 2020 [79]

Abbreviation: SEM, scanning electron microscope; FTIR, Fourier transform infrared spectroscopy spectra; AFM, atomic force microscopy.

## Data Availability

Not applicable.
